# Deciphering the stereo-specific catalytic mechanisms of *cis*-epoxysuccinate hydrolases producing L(+)-tartaric acid

**DOI:** 10.1016/j.jbc.2024.105635

**Published:** 2024-01-08

**Authors:** Sheng Dong, Jinsong Xuan, Yingang Feng, Qiu Cui

**Affiliations:** 1 CAS Key Laboratory of Biofuels, Shandong Provincial Key Laboratory of Synthetic Biology, Qingdao Institute of Bioenergy and Bioprocess Technology, Chinese Academy of Sciences, Qingdao, China; 2 Shandong Energy Institute, Qingdao, China; 3 Qingdao New Energy Shandong Laboratory, Qingdao, China; 4 University of Chinese Academy of Sciences, Beijing, China; 5 Department of Bioscience and Bioengineering, School of Chemistry and Biological Engineering, University of Science and Technology Beijing, Beijing, China

**Keywords:** epoxide hydrolase, *cis*-epoxysuccinate hydrolase, stereoselectivity, L(+)-tartaric acid, biocatalysis, crystal structure, catalytic mechanism

## Abstract

Microbial epoxide hydrolases, *cis*-epoxysuccinate hydrolases (CESHs), have been utilized for commercial production of enantiomerically pure L(+)- and D(−)-tartaric acids for decades. However, the stereo-catalytic mechanism of CESH producing L(+)-tartaric acid (CESH[L]) remains unclear. Herein, the crystal structures of two CESH[L]s in ligand-free, product-complexed, and catalytic intermediate forms were determined. These structures revealed the unique specific binding mode for the mirror-symmetric substrate, an active catalytic triad consisting of Asp-His-Glu, and an arginine providing a proton to the oxirane oxygen to facilitate the epoxide ring-opening reaction, which has been pursued for decades. These results provide the structural basis for the rational engineering of these industrial biocatalysts.

Epoxides are commonly encountered as intermediates in the catabolic pathway of various xenobiotics, and their oxirane moiety is a reactive chemical entity with the potential to interact with various biomolecules (1, 2). To mitigate this risk, organisms have evolved epoxide hydrolases (EHs) to convert these harmful intermediates into their corresponding vicinal diol (3). These biocatalysts often exhibit high levels of stereo- and regioselectivity, enabling the preparation of both epoxides and diols at high enantiomeric purity, which is desired for asymmetric synthesis (4, 5, 6). Despite their widespread distribution in nature, microbial EHs often preferred for preparative-scale biotransformations due to their abundance, superior efficiency, and ease of preparation (7, 8). Notably, recent efforts to modulate the catalytic properties of other EHs have successfully employed structure-based reshaping of the binding pocket or redesigning the active site (9, 10, 11, 12, 13).

The enantiomeric L(+)- and D(−)-tartaric acids (L-TA and D-TA), which feature two stereocenters, are widely recognized as valuable chiral synthons for the asymmetric synthesis of various chemicals and pharmaceuticals, as well as chiral selector (14, 15). Historically, L-TA was obtained from a by-product of the wine industry, which is limited by natural sources, while D-TA rarely exists in natural sources (16). As an alternative, microbial *cis*-epoxysuccinate hydrolases (CESHs) have been employed to produce large quantities of enantiomerically pure L-TA and D-TA (Fig. 1), with thousands of tons produced in factories each year (16, 17).

**Figure 1:**
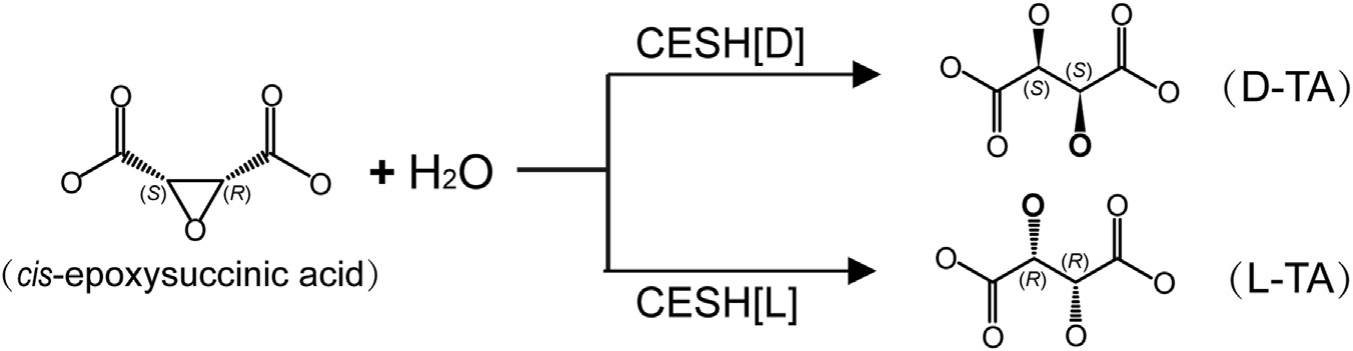
**Hydrolysis of *cis*-epoxysuccinic acidby CESH[L] or CESH[D] forms L(+)- or D(−)-tartaric acid, respectively.**

Although CESHs have been utilized for decades (since the 1970s) and efforts have been made to optimize the performance of these enzymes (16, 18, 19, 20), their catalytic mechanisms for their substrate selectivity, regio- and stereo-selectivity had not been fully elucidated because of the lack of high-resolution structures until 2018. In 2018, for the first time, we reported the crystal structure of a CESH that produces D-TA (CESH[D]) and elucidated the stereo-selective catalytic mechanism (21). The catalytic mechanism of CESH[L] has been studied using ^18^O tracing, homologous modeling, molecular docking, and mutagenesis analysis (22, 23), but the key residues for substrate binding and the mechanism of stereoselectivity are still not clear. Although several laboratories have dedicated their efforts to solving the crystal structures of CESH[L] more than 10 years ago (22, 24, 25, 26), there is no CESH[L] structure reported until now. The absence of substrate recognition and stereo-catalytic mechanism of CESH[L] limits the rational design and modifications.

The three best-characterized CESH[L]s to date are those from *Rhodococcus opacus* (*Rh*CESH[L]) (22, 27), *Nocardia tartaricans* (*No*CESH[L]) (28), and *Klebsiella* sp. BK-58 (*Kl*CESH[L]) (25), whose genes represent the only three CESH[L]s that have been cloned and sequenced. *Rh*CESH[L] and *No*CESH[L] share identical sequences, whereas *Kl*CESH[L] displays only 36% sequence identity with *Rh*CESH[L]. All these three CESH[L]s exhibit high sequence identity (>60%) with L-2-haloacid dehalogenases, which are members of the haloacid dehalogenase (HAD)-like superfamily (29, 30, 31) (Fig. S1). Studies have shown that both *Rh*CESH[L] and *Kl*CESH[L] adopt a two-step catalytic reaction mechanism by ^18^O-labeling studies (22, 23). The first step was proposed to be a nucleophilic attack on the carbon atom in the oxirane ring by residue D18 in *Rh*CESH[L] and residue D48 in *Kl*CESH[L], forming an enzyme-substrate intermediate and opening the ring with an unidentified proton donor. The second step was proposed to be the hydrolysis of the ester bond by an active water molecule activated by residues H190 and D193 in *Rh*CESH[L] and residues H221 and D224 in *Kl*CESH[L]. To elucidate the substrate recognition mechanism and stereo-catalytic mechanisms of CESH[L]s, we employed both *Rh*CESH[L] and *Kl*CESH[L] for crystallography in this study.

## Results

### Overall structures of CESH[L]s

**Figure 2:**
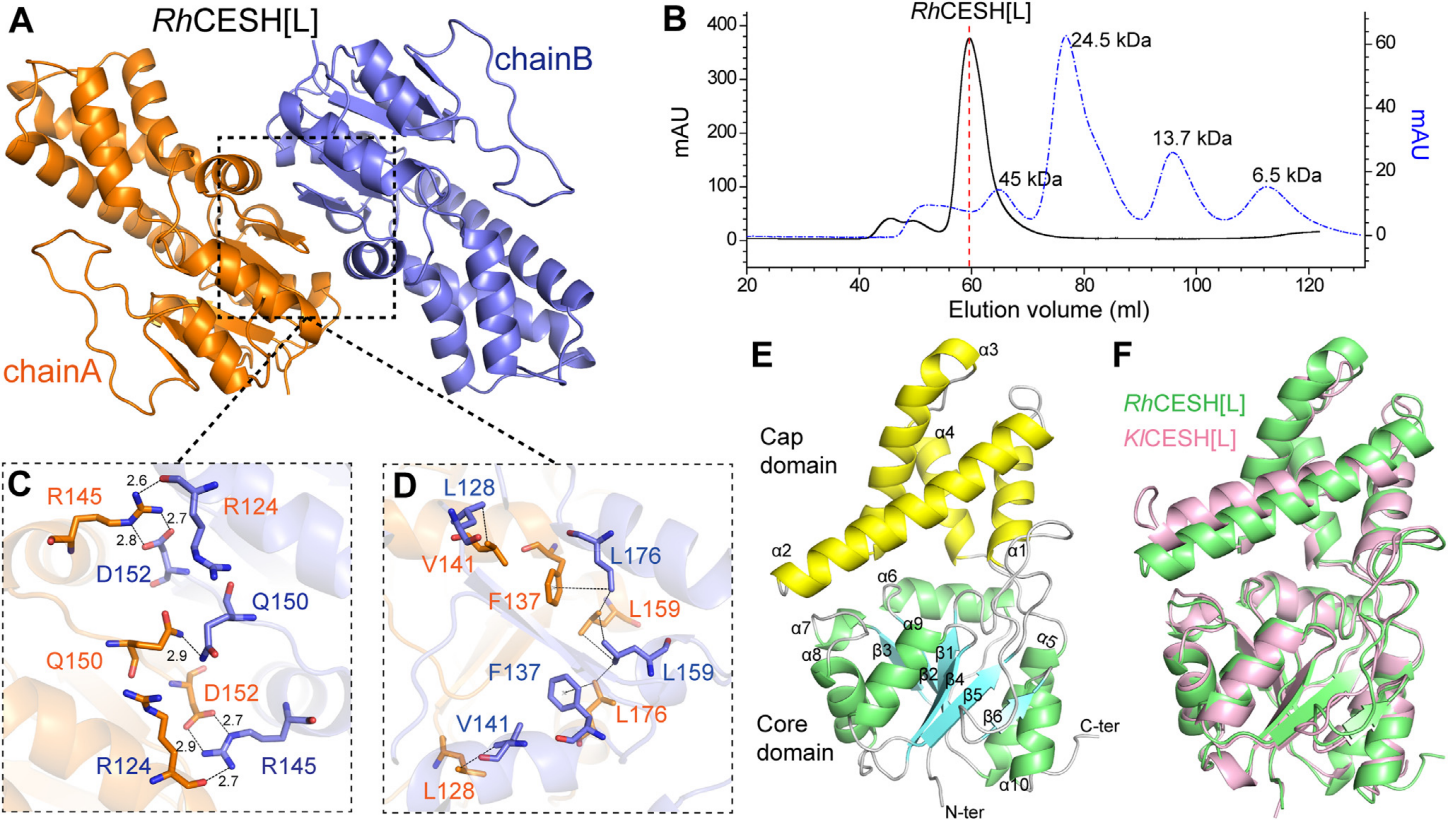
**The overall structure of CESH[L]s.***A*, a crystal asymmetric unit consisting of two molecules of *Rh*CESH[L]. *B*, gel filtration analysis of *Rh*CESH[L]. The theoretical molecular weight of recombinant *Rh*CESH[L] is approximately 29.3 kDa while its calculated apparent molecular weight in solution is approximately 52 kDa. The solid curve is the result of the gel filtration of *Rh*CESH[L] and the dashed curve is the result of the gel filtration of a molecular standard mixture containing ovalbumin (45.0 kDa), chymotrypsin (24.5 kDa), ribonuclease A (13.7 kDa), and aprotinin (6.5 kDa). *C*, analysis of key residues involved in hydrophilic interactions in *Rh*CESH[L] dimerization interface. *D*, key residues involved in hydrophobic interactions in *Rh*CESH[L] dimerization interface. *E*, the crystal structure of *Rh*CESH[L], with secondary structural elements labeled. The α-helices in the cap domain and the core domain are colored *yellow* and *green*, respectively. The β-strands are colored *cyan*. *F*, structural superimposition of *Rh*CESH[L] (*green*) and *Kl*CESH[L] (*pink*).

After crystal screening and optimization, crystal structures of *Rh*CESH[L] and *Kl*CESH[L] were successfully resolved at 1.94 Å and 2.02 Å, respectively (Table S1). Two molecules are present in a crystal asymmetric unit (Fig. 2*A*), and gel filtration experiments showed that both enzymes form homodimers in solution (Figs. 2*B* and S2). CESH[L] dimerization involves hydrophobic and hydrophilic regions, respectively. In *Rh*CESH[L], the key residues involved in hydrophilic interactions include R124, R145, Q150, and D152, while L128, F137, V141, L159, and L176 participate in hydrophobic interactions (Fig. 2, *C* and *D*). Furthermore, similar hydrophilic and hydrophobic interactions at the dimer interface are also observed in *Kl*CESH[L] (Fig. S2). Interestingly, an intermolecular disulfide bond forms symmetrically between C94 from two adjacent *Rh*CESH[L] molecules in the crystal structure; however, the aggregation state of *Rh*CESH[L] remains unchanged in the presence or absence of DTT, indicating that the disulfide bond forms due to crystal packing (Fig. S3).

Both enzymes exhibit the typical two-domain structure of the HAD-like superfamily, consisting of a cap domain with four helices, and a core domain with a Rossmann fold formed by alternating β-strands and α-helices (Fig. 2*E*) (32). The core domains of both *Rh*CESH[L] and *Kl*CESH[L] can be well superimposed (with an r.s.m.d value of 0.944 Å), while there are some notable differences in the cap domain (Fig. 2*F*). Furthermore, residues from the core domain surrounding the catalytic pocket in *Kl*CESH[L] can be superimposed well with those in *Rh*CESH[L], while some significant conformational differences are observed in the residues from the cap domain (Fig. S4).

### Polar substrate-binding pocket

The substrate of CESH[L]s, *cis*-epoxysuccinate (CES), is a small, highly hydrophilic, and mirror-symmetric molecule (16, 21). Despite this, CESH[L]s produce products with exceptionally high enantiomeric purity (22, 25), suggesting that the enzymes bind the substrate in a highly specific manner. To further unravel the mechanism of specific substrate recognition and stereo catalysis of CESH[L]s, we tried to get a structure of substrate-enzyme complex using several inactive mutants of *Rh*CESH[L] and *Kl*CESH[L] (Fig. S5).

**Figure 3:**
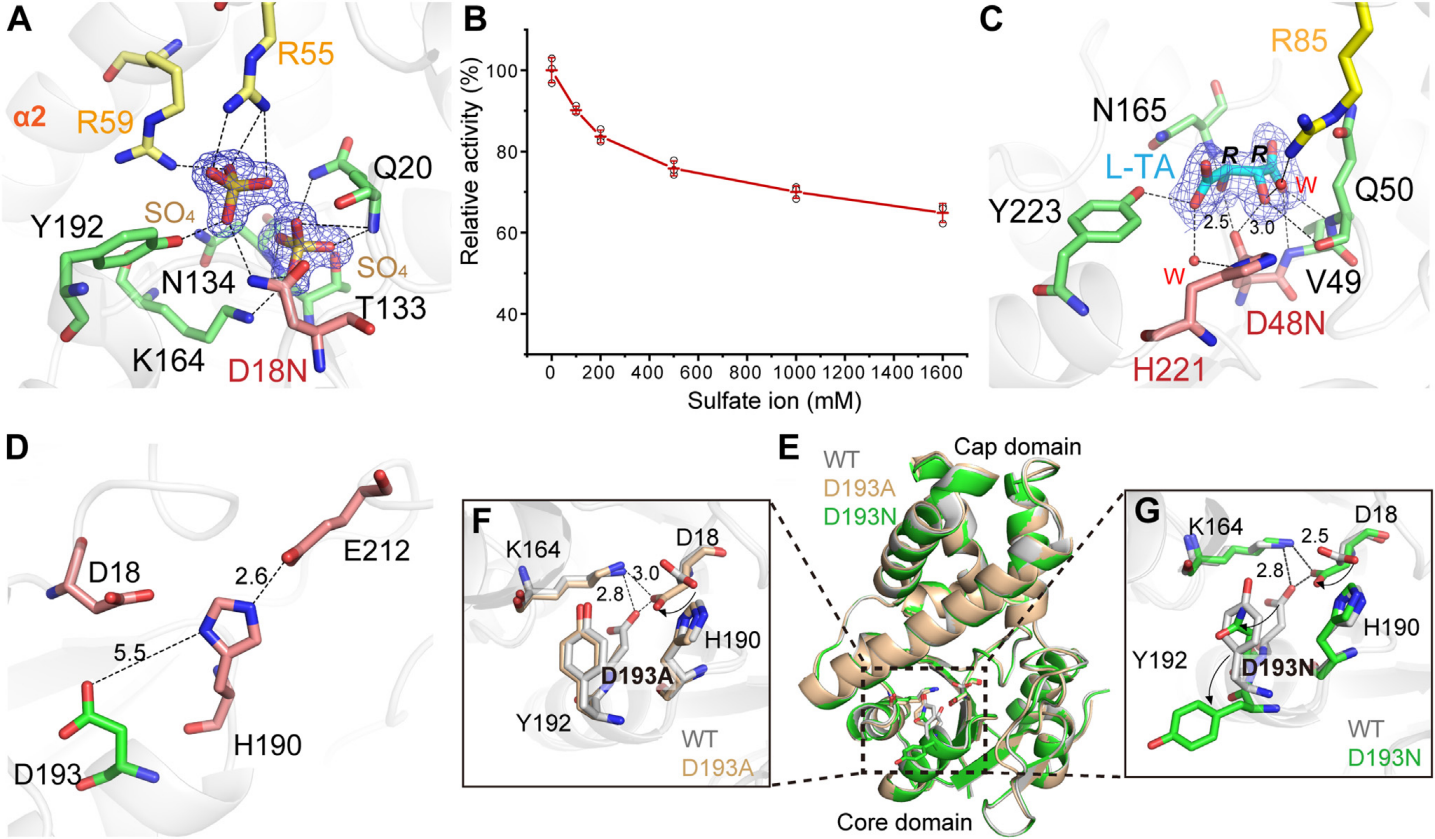
**Structural analysis of the substrate binding pocket of CESH[L]s.***A*, interactions of sulfate ions with *Rh*CESH[L]-D18N. *B*, effects of sulfate ions on *Rh*CESH[L] activity. The *open circles* indicate the individual data points of triplicate experiments. *C*, key amino acid residues interacted with L-TA in the active pocket of *Kl*CESH[L]-D48N/L-TA. The 2*mF*_o_-*DF*_c_ densities for sulfate ions and L-TA in *A* and *C*, respectively, are shown as blue mesh at the 1.0*σ* level. *D*, structural analysis of D193 in *Rh*CESH[L]. The residues in the core domain, cap domain, and catalytic triad are shown as *green*, *yellow*, and *pink sticks*, respectively, in figures *A*–*D*. *E*, comparative analysis of the overall structure of wild-type *Rh*CESH[L] with its D193A and D193N mutants. *F* and *G*, conformational analysis of key residues in the mutants *Rh*CESH[L]-D193A and *Rh*CESH[L]-D193N, respectively. Residues in the wild type *Rh*CESH[L] are depicted in grey sticks, while those in *Rh*CESH[L]-D193A and *Rh*CESH[L]-D193N are depicted in wheat and *green sticks*, respectively. *Dashed lines* represent possible interactions, and distances are shown in angstroms. The distances of unlabeled dash lines are less than 3.5 Å.

*Rh*CESH[L]-D18N was first used to capture the complex structure of CESH[L]s and CES because D18 was proposed to be the nucleophilic attacking residue in previous studies (22). However, sulfate ions, from the crystallizing solutions, were observed in the catalytic pockets of *Rh*CESH[L] after the co-crystallization with CES (Fig. 3*A*, Table S1). The residues involved in sulfate ion binding, including D18N, Q20, T133, N134, K164, and Y192 from the core domain, as well as R55 and R59 from the cap domain, formed a polar substrate binding pocket that was well-suited for binding small, highly hydrophilic molecules (Fig. 3*A*). Activity assays revealed a decrease in *Rh*CESH[L] activity with increasing sulfate ion concentration, indicating that sulfate ions can compete for the binding site during catalysis (Fig. 3*B*).

We also constructed an inactive mutant, *Kl*CESH[L]-D48N, to obtain the enzyme-substrate structure because D48 was proposed to be the first step catalytic residue (23). A product molecule, L-TA, was found in the substrate-binding site of *Kl*CESH[L]-D48N, which was present in the crystallizing solutions at a concentration of 0.2 M (Table S1, Fig. S6*A*). One carboxyl group of the L-TA is fixed by N165 and Y223, and the other carboxyl group is mainly fixed by the nitrogen atom of the main chain of V49 and Q50. The distances between the L-TA two hydroxyl oxygen atoms and the side chain oxygen atom of N48 are ∼2.5 to 3.0 Å, supporting that D48 is the nucleophilic residue in *Kl*CESH[L]. However, D48 has similar distances to the two hydroxyl oxygen atoms of L-TA, making it difficult to determine which hydroxyl group is derived from the oxygen atom of the epoxy during the reaction (Fig. 3*C*).

Although the previously proposed catalytic triad of *Rh*CESH[L] consists of D18, H190, and D193 (22), D193 should not be the catalytic residue in the catalytic triad because D193 does not form direct interaction with H190 in the solved *Rh*CESH[L] structure (Fig. 3*D*). To elucidate the mechanism of inactivation caused by D193 mutation, we determined the crystal structure of *Rh*CESH[L]-D193A and *Rh*CESH[L]-D193N (Table S1). Comparison of these structures with the wild-type *Rh*CESH[L] structure shows that these mutations did not lead to significant changes in the overall structure (with an r.m.sd less than 0.24 Å) (Fig. 3*E*). However, the mutations of D193 disrupted a salt bridge with K164 and resulted in a significant conformation change of D18 which subsequently formed a new salt bridge with K164 (Fig. 3, *F* and *G*). This explains why the mutation of D193 caused the loss of enzyme catalytic activity (22, 23).

### Stereo-specific catalytic mechanisms

**Figure 4:**
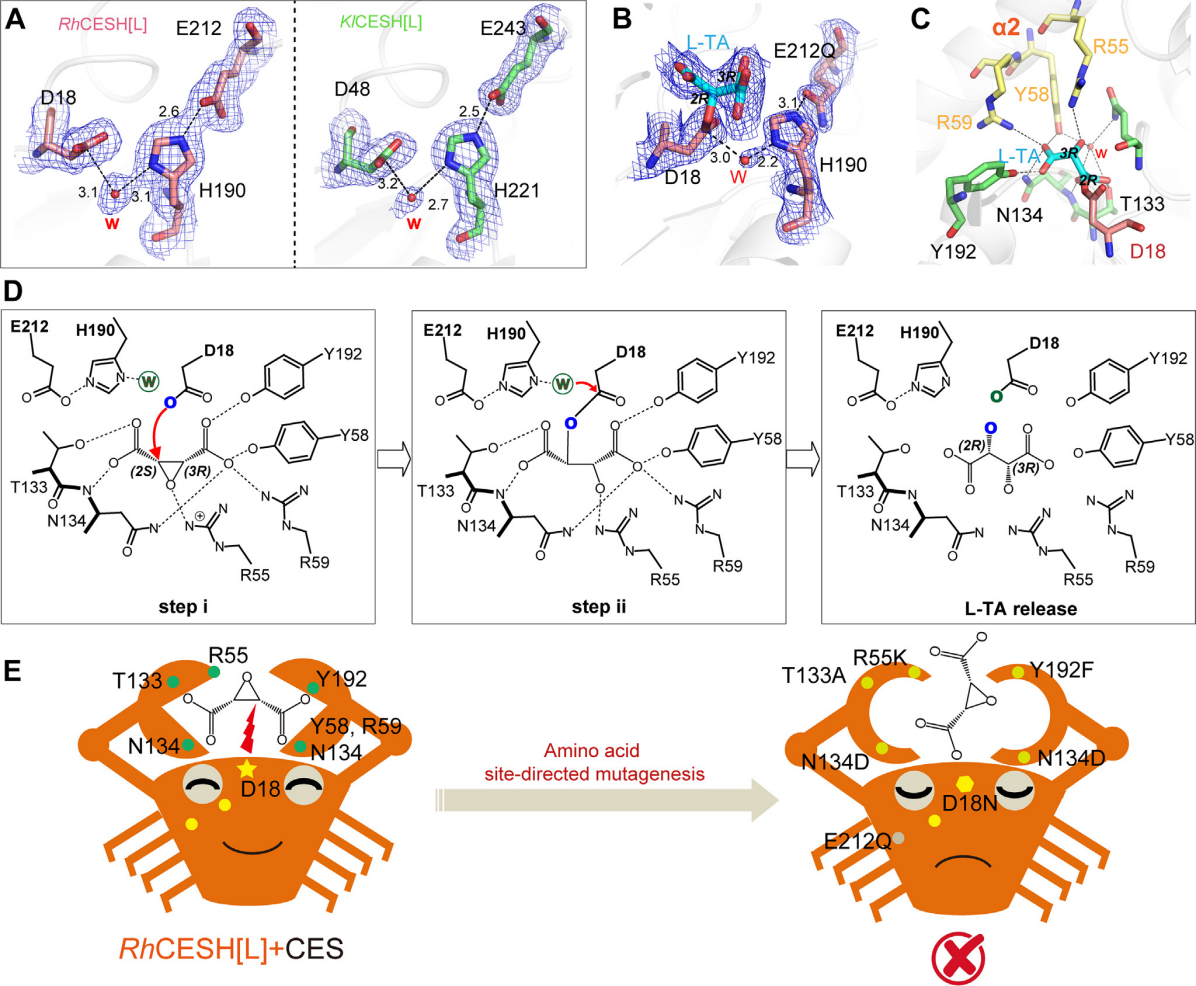
**Catalytic mechanism of CESH[L]s.***A*, catalytic triad and active water in *Rh*CESH[L] and *Kl*CESH[L]. *B*, catalytic triad, active water, and ester intermediate in *Rh*CESH[L]-E212Q/L-TA structure. *C*, residues interacting with the ligand in *Rh*CESH[L]-E212Q/L-TA. The residues in the core domain, cap domain, and catalytic triad are shown as *green*, *yellow*, and *pink sticks*, respectively. *D*, schematic diagram of the proposed two-step catalytic mechanism of CESH[L]s. *E*, caricature view of the stereo-catalytic mechanism of CESH[L]s. The 2*mF*_o_-*DF*_c_ densities for residues, ligands, and water are shown as blue mesh at the 1.0*σ* level. *Dashed lines* represent possible interactions, and distances are shown in angstroms. The distances of unlabeled *dash lines* are less than 3.5 Å.

Careful analysis of the obtained CESH[L] structures revealed a potential active catalytic triad consisting of D18, H190, and E212, along with the active water molecule in *Rh*CESH[L] while the corresponding possible catalytic triad in *Kl*CESH[L] consists of D48, H221, and E243 (Fig. 4*A*). Combined with the knowledge of the CESH[L] catalytic mechanism in previous studies (22, 23, 28), we hypothesize that D18 in *Rh*CESH[L] and D48 in *Kl*CESH[L] serve as nucleophilic residues, responsible for initiating the nucleophilic attack during the first step of the enzymatic reaction, while E212 in *Rh*CESH[L] and E243 in *Kl*CESH[L] function as catalytic residues in the second step reaction, along with the histidine residues to active the water for the hydrolysis of ester bond in the intermediate.

In light of this hypothesis, we constructed another inactive mutant, *Rh*CESH[L]-E212Q, which was expected to halt the catalytic reaction at the second step and create an enzyme-substrate ester intermediate. *Rh*CESH[L]-E212Q was cocrystallized with CES and the structure was solved at 2.20 Å. As expected, D18 formed an ester intermediate with the ligand in the structure. The D18/L-TA, E212Q, H190, and the active water were all with unambiguous electron densities (Figs. 4*B* and S6*B*). The carboxyl substituent on C2 of the substrate is bound by residue T133 and N134 *via* hydrogen bonds, as well as a water-mediated hydrogen bond networks; while the cap domain’s Y58 and R59, along with N134 and Y192 from the core domain, bound the carboxyl group on C3 *via* polar interactions (Fig. 4*C*). Interestingly, residue R55 forms a hydrogen bond with the C3 hydroxyl group (Fig. 4*C*), and the substitution of R55 with Lys leads to a loss of enzyme activity, along with a significant increase in the *K*_*m*_ value (22). This suggests that R55 should play a critical role in positioning the oxygen of the epoxide, as well as provide a proton to the oxirane oxygen atom to form a hydroxyl that facilitates the epoxide ring-opening reaction.

Based on the structural analysis, we propose the two-step stereo-catalytic mechanism of *Rh*CESH[L]: (i) CES is anchored by polar residues surrounding the binding pocket, which expose the C2 atom to nucleophilic attack; the nucleophilic residue D18 attacks the C2 atom of CES in a stereo-specific manner to form an enzyme-substrate ester intermediate, while protonated R55 provides a proton to the oxirane oxygen atom to form a hydroxyl group; and (ii) the E212-H190 diad activates water to attack the ester bond, leading to the release of L-TA (Fig. 4*D*).

## Discussion

In the structure of *Rh*CESH[L]-E212Q/L-TA, we observed a significant movement of the α2 helix in the cap domain, containing Y58 and R59 that directly interact with the substrate, toward the core domain (Fig. S7). Conformational changes are commonly observed in enzyme catalysis following substrate binding (33, 34). However, the binding of non-catalytic ligands, including sulfate ions and products L-TA, did not cause significant structural changes (Fig. S7).

The two-step catalytic mechanisms observed in CESH[L]s are also commonly seen in L-2-haloacid dehalogenase, which is a representative enzyme of the HAD superfamily (22, 23). However, CESH[L]s have a different substrate type from L-2-haloacid dehalogenase, as they act on epoxides and haloacids, respectively. This reflects the adaptive evolution of enzymes from a common ancestor that have become specialized for different substrates. In contrast, many well-known EHs do not share significant sequence similarity with CESH[L]s (35), suggesting a divergent evolution of these proteins. Notably, CESH[L]s adopt the Rossmann fold structure in their core domain, which is distinct from the α/β hydrolase fold structure seen in many other EHs (35).

Due to the mirror-symmetric nature of CES resulting from the two carboxyl substituent groups, the CESH[L]'s substrate binding pocket has evolved to resemble crab claws, capable of binding a carboxyl group individually. One side of the crab claw is formed by T133 and N134, while the other side consists of residues Y58, R59, N134, and Y192 (Fig. 4*E*). Previous research has demonstrated that site-directed mutations in these amino acids lead to a loss of enzymatic activity (22, 25). Through our structural analysis, we have elucidated the crucial role of these amino acids in anchoring the substrate during the catalytic process (Fig. 4*E*). Their strategic positioning within the binding pocket ensures precise substrate recognition and enables efficient catalysis.

As a biocatalyst used for dozens of years, CESHL[L] has been engineered for better performance in both stability and activity. For example, directed evolution has been successfully used to enhance the thermostability and activity of CESH[L] (24, 36). Various immobilization techniques have been adopted to enhance the stability of the enzyme or the whole-cell catalyst of CESH[L] (19, 37, 38). Recently, we developed a bacterial surface-display system of CESH[L] with high stability and activity (39). The structures and the catalytic mechanism of CESH[L] obtained in the current paper provide the basis for future rational engineering of CESH[L] to achieve better biocatalysts.

In conclusion, we determined the crystal structures of two representative CESH[L]s that are responsible for the industrial production of L(+)-tartaric acid, encompassing the enzyme in its free form, in complex with the product, and as catalytic intermediates. The obtained structures reveal the unique and specific binding modes employed by CESH[L]s. Furthermore, we identified their catalytic triad consisting of Asp-His-Glu, along with the involvement of an arginine residue in facilitating the opening of the epoxy ring by protonating the epoxide oxygen. By deciphering the catalytic mechanisms of CESH[L]s, our study lays the foundation for rational enzyme designs, leading to new potential applications for these long-utilized biocatalysts.

## Experimental procedures

### Protein expression and purification

Site-directed mutations were constructed using overlapping PCR (Table S2). For efficient heterologous expression and protein crystallization, *Rh*CESH[L] and its mutants lacking the first ten residues were amplified and cloned into pET28a between the *Nde* I and *EcoR* I restriction sites, while *Kl*CESH[L] and its mutants lacking the first 40 residues were amplified and cloned into pET30a between the *Nde* I and *Xho* I restriction sites, respectively. The proteins with His_6_-tag were expressed in *Escherichia coli* strain BL21(DE3) and induced with 0.2 mM isopropyl β-D-1-thiogalactopyranoside (IPTG) at 16 °C for 18 h. The cells were harvested by centrifugation and resuspended into binding buffer (50 mM Tris-HCl, pH 8.0, 500 mM NaCl, 10 mM imidazole). The cells were then lysed by ultrasonication and centrifugated at 15,000*g* for 30 min. The supernatant fluids were applied onto a Ni^2+^ Sepharose HP column (GE Healthcare), and the target proteins were eluted by elution buffer (50 mM Tris-HCl, pH 8.0, 500 mM NaCl, 250 mM imidazole). The proteins were further purified by gel filtration with a Superdex 75 column (GE Healthcare) with a buffer containing 10 mM Tris-HCl, pH 8.0, and 100 mM NaCl. The purity of the target proteins was judged to be >95% by polyacrylamide gel electrophoresis. The protein concentration was determined by the ultraviolet absorption at 280 nm using a theoretical molar extinction coefficient. and stored at −80 °C in a storage buffer consisting of 10 mM Tris-HCl, pH 8.0, and 100 mM NaCl for subsequent use.

### Enzyme activity measurement

The enzyme activity of CESH[L] was measured as we reported previously (18, 19, 21). Briefly, 0.02 ml of enzyme solution was added to a mixture of 0.98 ml of 1.0 M disodium *cis*-epoxysuccinate in 200 mM sodium phosphate buffer, pH 8.0. The solutions were incubated at 37 °C for 20 min and the reactions were terminated by adding 0.4 ml of 1.0 M H_2_SO_4_. The tartaric acid generated in the reactions was measured using the ammonium metavanadate method. Specifically, 1 ml of 1% (w/v) ammonium metavanadate was added to the reaction solution, which was then diluted to 10 ml. After waiting for 5 min, the absorbance at 480 nm was measured using a Synergy HT Multi-mode microplate reader (BioTek Instruments, Inc). The tartaric acid concentration in the reaction solution was calculated according to the standard curve obtained from tartaric acid solutions of different concentrations.

### Crystallization, data collection, and structure determination

The purified proteins for crystallization were concentrated to approximately 20 mg/ml in 10 mM Tris-HCl (pH 8.0) and 100 mM NaCl. Crystals were obtained using sitting-drop vapor diffusion for screening and hanging-drop vapor diffusion for optimization at 18 °C. To obtain crystals of the CESH[L]/CES complex, CESH[L] mutants were mixed with CES at a 1:50 molar ratio. High-quality crystals were obtained under the conditions shown in Table S1. All of the crystals used for data collection were cryoprotected by soaking in a well solution supplemented with 20% (v/v) glycerol for 10 s and then flash-cooled in liquid nitrogen. X-ray diffraction data were collected on the BL17U1, BL10U1, or BL19U1 beamline at the Shanghai Synchrotron Radiation Facility (SSRF) (40, 41).

Data indexing, integration, and scaling were conducted using HKL3000 or XDS (42, 43). The structures of *Rh*CESH[L] and *Kl*CESH[L] were determined by molecular replacement using the PHENIX program suite and the PDB 3UMC as a search model. Their structures were built automatically by Autobuild in the PHENIX program package (44). Then the wild-type CESH[L]s were used as the search model for resolving their mutant structures. Refinement of the structure was performed using the programs COOT and PHENIX (45). All the final models were evaluated using MolProbity (46), and data collection and refinement statistics were shown in Table S1. All molecular graphics were created with PyMOL (http://www.pymol.org).

## Data availability

The atomic coordinates and structure factors have been deposited in the Protein Data Bank (www.pdb.org) with PDB ID codes 8WBK, 8WBL, 8WBM, 8WBN, 8WBO, 8WBP, 8WBQ, 8WBR, 8WBS, and 8WBT.

## Supporting information

This article contains supporting information.

## Conflict of interest

The authors declare that they have no conflicts of interest with the contents of this article.
