# Supplementary material for: Deciphering the stereo-specific catalytic mechanisms of cis-epoxysuccinate hydrolases producing L(+)-tartaric acid
Source: J Biol Chem. 2024 Jan 8;300(2):105635. doi: 10.1016/j.jbc.2024.105635 (PMC10869282; doi:10.1016/j.jbc.2024.105635)
Supplement: Supporting Information [file mmc1.docx]

Supporting information

Deciphering the stereo-specific catalytic mechanisms of *cis*-epoxysuccinate hydrolases producing L(+)-tartaric acid

Sheng Dong^1,2,3,4^, Jinsong Xuan^5^, Yingang Feng^1,2,3,4^* and Qiu Cui^1,2,3,4*^

*^1^CAS Key Laboratory of Biofuels, Shandong Provincial Key Laboratory of Synthetic Biology, Qingdao Institute of Bioenergy and Bioprocess Technology, Chinese Academy of Sciences, Qingdao 266101, China*

*^2^Shandong Energy Institute, Qingdao 266101, China*

*^3^Qingdao New Energy Shandong Laboratory, Qingdao 266101, China*

*^4^University of Chinese Academy of Sciences, Beijing 100049, China*

*^5^Department of Bioscience and Bioengineering, School of Chemistry and Biological Engineering, University of Science and Technology Beijing, 30 Xueyuan Road, Beijing 100083, China*

**Correspondence: Yingang Feng, Fengyg@qibebt.ac.cn (Y. Feng) and Qiu Cui, cuiqiu@qibebt.ac.cn (Q.Cui).*

**Table S1**. Crystallographic data collection and refinement statistics for CESH[L]s.

|  | *Rh*CESH[L] | *Rh*CESH[L] | *Rh*CESH[L]-D193A |
| --- | --- | --- | --- |
| **PDB code** | 8WBK | 8WBL | 8WBM |
| **Ligands** | Free | Sulfate ion | Sulfate ion |
| **Crystallization** | 0.2 M potassium sodium tartrate tetrahydrate, 0.1 M sodium citrate tribasic dihydrate pH5.6, 2.0 M ammonium sulfate | 0.2 M potassium sodium tartrate tetrahydrate, 0.1 M sodium citrate tribasic dihydrate pH5.6, 2.0 M ammonium sulfate | 0.05 M ammonium sulfate, 0.05 M Bis-tris pH6.5, 30% pentaerythritol ethoxylate |
| **Data collection**^a^ |  |  |  |
| Space group | P1 | P1 | P1 |
| *a,b,c* (Å)  *α**, β, γ* (°) | 42.13, 55.62, 64.20  67.34, 75.80, 75.80 | 43.53, 59.35, 64.57  69.46, 73.64, 82.65 | 44.26, 59.49, 64.37  68.23, 75.27, 83.44 |
| Wavelength (Å) | 0.979 | 0.979 | 0.979 |
| Resolution (Å) | 58.40-2.15 (2.21-2.15) | 55.55-1.94 (2.05-1.94) | 58.18-2.06 (2.12-2.06) |
| Unique reflections | 26753 (2005) | 37230 (5677) | 34339 (2566) |
| Completeness (%) | 95.5 (96.7) | 86.7 (90.4) | 94.7 (94.4) |
| Redundancy | 2.0 (2.0) | 3.6 (3.6) | 3.7 (3.8) |
| *Mean I/sigma (I)* | 8.5 (3.1) | 12.9 (3.2) | 8.6 (4.1) |
| CC (1/2) | 0.993 (0.964) | 0.998 (0.940) | 0.986 (0.935) |
| R_merge_^b^ | 0.064 (0.098) | 0.047 (0.383) | 0.095 (0.235) |
| **Refinement** |  |  |  |
| *R_work_/R_free_* (%) | 20.81/25.69 | 19.05/22.06 | 20.06/23.74 |
| **No. atoms** |  |  |  |
| Protein | 3740 | 3730 | 3740 |
| Ligand | 5 | 20 | 10 |
| water | 154 | 117 | 438 |
| **B-factors** |  |  |  |
| Average B-factor | 43.30 | 45.79 | 28.20 |
| Proteins | 43.30 | 45.55 | 27.24 |
| Ligand | 48.77 | 78.73 | 25.59 |
| Solvent | 43.06 | 48.01 | 36.44 |
| **r.m.s.d.** |  |  |  |
| Bond length (Å) | 0.008 | 0.019 | 0.008 |
| Bond angles (°) | 1.03 | 1.69 | 0.88 |
| **MolProbity score** | 1.36 | 1.19 | 1.38 |
| **Ramachandran statistics** | | |  |
| Favored (%) | 99.57 | 99.79 | 99.57 |
| Outliers (%) | 0.00 | 0.00 | 0.00 |

a. Values in parentheses refer to data in the highest-resolution shell.

b. R*_merge_*=Σ*_hkl_*Σ*_i_*|*I*(*hkl*)*_i_*-<*I(hkl)*>|/Σ_hkl_Σ_i_<*I(hkl)_i_*>, where *I* is the observed intensity, <*I(hkl)*> represents the average intensity, and *I(hkl)_i_* represents the observed intensity of each unique reflection.

**Table S1**. Crystallographic data collection and refinement statistics for CESH[L]s (continued).

|  | *Rh*CESH[L]-D193N | *Rh*CESH[L]-D18N | *Rh*CESH[L]-E212Q |
| --- | --- | --- | --- |
| **PDB code** | 8WBN | 8WBO^c^ | 8WBP |
| **Ligands** | Sulfate ion | Sulfate ion | Free |
| **Crystallization** | 0.2 M ammonium sulfate, 0.1 M Bis-tris, pH6.5, 25%PEG 3350 | 0.2 M Lithium sulfate monohydrate, 0.1 M Bis-Tris, pH 6.5, 26% PEG3350 | 0.2 M sodium citrate tribasic, 0.2 M potassium acetate, 20% PEG3350 |
| **Data collection**^a^ |  |  |  |
| Space group | P1 | C2 | P1 |
| *a,b,c* (Å)  *α, β, γ* (°) | 43.40, 58.55, 64.09  69.88, 73.55, 82.69 | 63.80, 59.52, 68.68  90.00, 108.41, 90.00 | 43.16, 57.93, 62.96  68.30, 74.57, 83.03 |
| Wavelength (Å) | 0979 | 0.979 | 0.979 |
| Resolution (Å) | 48.55-2.50 (2.60-2.50) | 50.00-1.58 (1.61-1.58) | 53.80-1.87 (1.98-1.87) |
| Unique reflections | 17521 (1990) | 33520 (1679) | 42099 (6226) |
| Completeness (%) | 89.1 (89.7) | 99.9 (99.9) | 93.3 (85.3) |
| Redundancy | 1.9 (1.9) | 6.7 (6.4) | 3.4 (3.1) |
| *Mean I/sigma (I)* | 4.3 (1.6) | 39 (4.4) | 12.2 (3.6) |
| CC (1/2) | 0.979 (0.824) | 0.998(0.931) | 0.996 (0.954) |
| R_merge_^b^ | 0.135 (0.540) | 0.045 (0.411) | 0.048 (0.239) |
| **Refinement** |  |  |  |
| *R_work_/R_free_* (%) | 21.06/26.56 | 15.06/17.94 | 16.89/19.72 |
| **No. atoms** |  |  |  |
| Protein | 3724 | 1873 | 3727 |
| Ligand | 10 | 10 | 0 |
| water | 13 | 251 | 329 |
| **B-factors** |  |  |  |
| Average B-factor | 64.27 | 19.72 | 40.00 |
| Proteins | 64.34 | 18.16 | 39.42 |
| Ligand | 51.38 | 15.68 | - |
| Solvent | 54.06 | 31.50 | 46.55 |
| **r.m.s.d.** |  |  |  |
| Bond length (Å) | 0.003 | 0.006 | 0.008 |
| Bond angles (°) | 0.061 | 0.88 | 1.06 |
| **MolProbity score** | 1.22 | 0.81 | 1.52 |
| **Ramachandran statistics** | | |  |
| Favored (%) | 99.57 | 100.00 | 99.57 |
| Outliers (%) | 0.00 | 0.00 | 0.00 |

a. Values in parentheses refer to data in the highest-resolution shell.

b. R*_merge_*=Σ*_hkl_*Σ*_i_*|*I*(*hkl*)*_i_*-<*I(hkl)*>|/Σ_hkl_Σ_i_<*I(hkl)_i_*>, where *I* is the observed intensity, <*I(hkl)*> represents the average intensity, and *I(hkl)_i_* represents the observed intensity of each unique reflection.

c. The structure has a relatively high number of RSRZ outliers in the PDB validation report because of the poor density of some regions in the cap domain.

**Table S1**. Crystallographic data collection and refinement statistics for CESH[L]s (continued).

|  | *Rh*CESH[L]-E212Q | | *Kl*CESH[L] | | *Kl*CESH[L]-D48N | |
| --- | --- | --- | --- | --- | --- | --- |
| **PDB code** | 8WBQ | | 8WBR | | 8WBS^c^ | |
| **Ligands** | L(+)-tartaric acid | | Free | | Sulfate ion | |
| **Crystallization** | 0.2 M sodium citrate tribasic, 0.2 M potassium acetate, 20% PEG3350 | | 0.2 M Potassium formate, 20% PEG3350 | | 0.2 M Lithium sulfate monohydrate, 0.1 M Bis-Tris, pH 6.5, 25% PEG3350 | |
| **Data collection**^a^ |  | |  | |  | |
| Space group | P1 | | P2_1_2_1_2_1_ | | P2_1_2_1_2 | |
| *a,b,c* (Å)  *α, β, γ* (°) | 40.64, 53.89, 61.96  67.07, 75.84, 75.97 | | 66.30, 84.75, 92.85  90.00, 90.00, 90.00 | | 119.85, 94.43, 99.60  90.00, 90.00, 90.00 | |
| Wavelength (Å) | 0.979 | | 0.979 | | 0.979 | |
| Resolution (Å) | 56.25-2.20 (2.26-2.20) | | 38.55-2.02 (2.07-2.02) | | 119.85-2.03 (2.08-2.03) | |
| Unique reflections | 23063 (1694) | | 34818 (2517) | | 73725 (5375) | |
| Completeness (%) | 98.2 (97.4) | | 99.5 (99.0) | | 100.0 (100.0) | |
| Redundancy | 3.4 (2.9) | | 6.2 (5.5) | | 13.4 (12.2) | |
| *Mean I/sigma (I)* | 5.8 (3.3) | | 11.3 (2.4) | | 14.9 (2.2) | |
| CC (1/2) | 0.926 (0.264) | | 0.997 (0.817) | | 0.999 (0.739) | |
| R_merge_^b^ | 0.219 (1.089) | | 0.077 (0.848) | | 0.100 (1.343) | |
| **Refinement** |  | |  | |  | |
| *R_work_/R_free_* (%) | 18.25/23.95 | | 20.82/23.56 | | 18.35/21.47 | |
| **No. atoms** |  | |  | |  | |
| Protein | 3795 | | 3718 | | 7349 | |
| Ligand | 18 | | 1 | | 42 | |
| water | 116 | | 245 | | 357 | |
| **B-factors** |  | |  | |  | |
| Average B-factor | 36.73 | | 48.33 | | 54.73 | |
| Proteins | 36.81 | | 48.38 | | 54.93 | |
| Ligand | 45.08 | | 60.04 | | 69.59 | |
| Solvent | 33.07 | | 47.59 | | 48.96 | |
| **r.m.s.d.** |  | |  | |  | |
| Bond length (Å) | 0.006 | | 0.004 | | 0.008 | |
| Bond angles (°) | 0.66 | | 0.97 | | 1.22 | |
| **MolProbity score** | 1.65 | | 1.14 | | 1.25 | |
| **Ramachandran statistics** | |  | |  | |  |
| Favored (%) | 99.79 | | 98.29 | | 98.03 | |
| Outliers (%) | 0.00 | | 0.00 | | 0.00 | |

a. Values in parentheses refer to data in the highest-resolution shell.

b. R*_merge_*=Σ*_hkl_*Σ*_i_*|*I*(*hkl*)*_i_*-<*I(hkl)*>|/Σ_hkl_Σ_i_<*I(hkl)_i_*>, where *I* is the observed intensity, <*I(hkl)*> represents the average intensity, and *I(hkl)_i_* represents the observed intensity of each unique reflection.

c. The structure has a relatively high number of RSRZ outliers in the PDB validation report because of the poor density of some regions in the cap domain of chains C and D.

**Table S1**. Crystallographic data collection and refinement statistics for CESH[L]s (continued).

|  | *Kl*CESH[L]-D48N | |
| --- | --- | --- |
| **PDB code** | 8WBT^c^ | |
| **Ligands** | L(+)-tartaric acid | |
| **Crystallization** | 0.2 M ammonium tartrate dibasic, pH7.0, 20% PEG3350 | |
| **Data collection**^a^ |  | |
| Space group | P2_1_2_1_2 | |
| *a,b,c* (Å)  *α, β, γ* (°) | 119.62, 93.15, 101.64  90.00, 90.00, 90.00 | |
| Wavelength (Å) | 0.979 | |
| Resolution (Å) | 30.29-2.05 (2.10-2.05) | |
| Unique reflections | 71832 (5212) | |
| Completeness (%) | 99.9 (99.6) | |
| Redundancy | 6.6 (6.4) | |
| *Mean I/sigma (I)* | 11.4 (2.1) | |
| CC (1/2) | 0.997 (0.714) | |
| R_merge_^b^ | 0.114 (0.859) | |
| **Refinement** |  | |
| *R_work_/R_free_* (%) | 17.11/21.04 | |
| **No. atoms** |  | |
| Protein | 7503 | |
| Ligand | 32 | |
| water | 604 | |
| **B-factors** |  | |
| Average B-factor | 43.21 | |
| Proteins | 43.19 | |
| Ligand | 51.84 | |
| Solvent | 43.09 | |
| **r.m.s.d.** |  | |
| Bond length (Å) | 0.008 | |
| Bond angles (°) | 1.15 | |
| **MolProbity score** | 1.18 | |
| **Ramachandran statistics** | |  |
| Favored (%) | 98.40 | |
| Outliers (%) | 0.00 | |

a. Values in parentheses refer to data in the highest-resolution shell.

b. R*_merge_*=Σ*_hkl_*Σ*_i_*|*I*(*hkl*)*_i_*-<*I(hkl)*>|/Σ_hkl_Σ_i_<*I(hkl)_i_*>, where *I* is the observed intensity, <*I(hkl)*> represents the average intensity, and *I(hkl)_i_* represents the observed intensity of each unique reflection.

c. The structure has a relatively high number of RSRZ outliers in the PDB validation report because of the poor density of some regions in the cap domain of chain D.

**Table S2.** Primers used in this study. Underlined sequences indicate the restriction sites.

| Primers | Sequence (5’-3’) | Usage/comments |
| --- | --- | --- |
| CESHL-RF | GGAATTCCATATGCAGTTCCGTGCTCTGCTGTTC |  |
| CESHL-RR | CCGGAATTCTTATTAGTCGATACCAGCG |  |
| R-D18NF | GGAATTCCATATGCAGTTCCGTGCTCTGCTGTTCAACGTTCAGG |  |
| R-D18NR | CCGGAATTCTTATTAGTCGATACCAGCG |  |
| R-D193AF | CTCACGCTTACGCCCTGGAAGCTG |  |
| R-D193AR | CAGCTTCCAGGGCGTAAGCGTGAG |  |
| R-E212QF | CGTCCGCTGCAATACGGTCCGAC |  |
| R-E212QR | GTCGGACCGTATTGCAGCGGACG |  |
| CESHL-KF | GGACTTCCATATGGGTCTGAAAGCTCTGTTCTTC |  |
| CESHL-KR | CCGCTCGAGAGCACCCAGCATACCAGCCAG |  |
| K-D48NF | GGACTTCCATATGGGTCTGAAAGCTCTGTTCTTCAACGTTCAGGGT |  |
| K-D48NR | CCGCTCGAGAGCACCCAGCATACCAGCCAG |  |


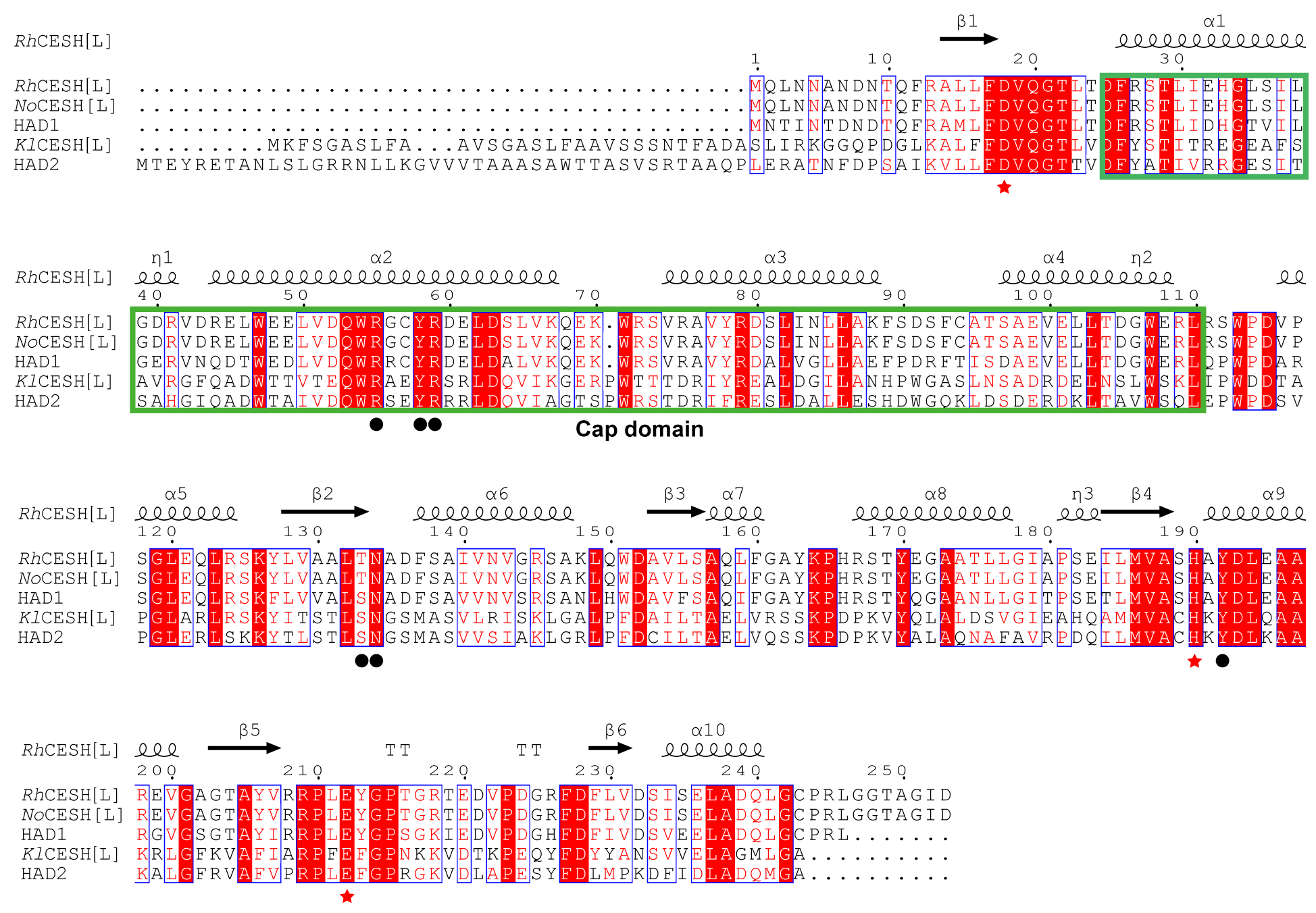


**Figure S1.** Sequence alignment for CESH[L]s with L-2-haloacid dehalogenase. Secondary structural elements of the *Rh*CESH[L] are shown above the alignment. The catalytic triad residues are indicated by red stars. Conserved residues that are responsible for CES binding are indicated by black circles. The residues that are composed of the cap domain are shown in green boxes. *Rh*CESH[L], *No*CESH[L], and *Kl*CESH[L] are in this study, HAD1 is a haloacid dehalogenase from *Rhodococcus* sp. ACPA4, and HAD2 is a haloacid dehalogenase from *Methylobacterium* sp. J-090 .


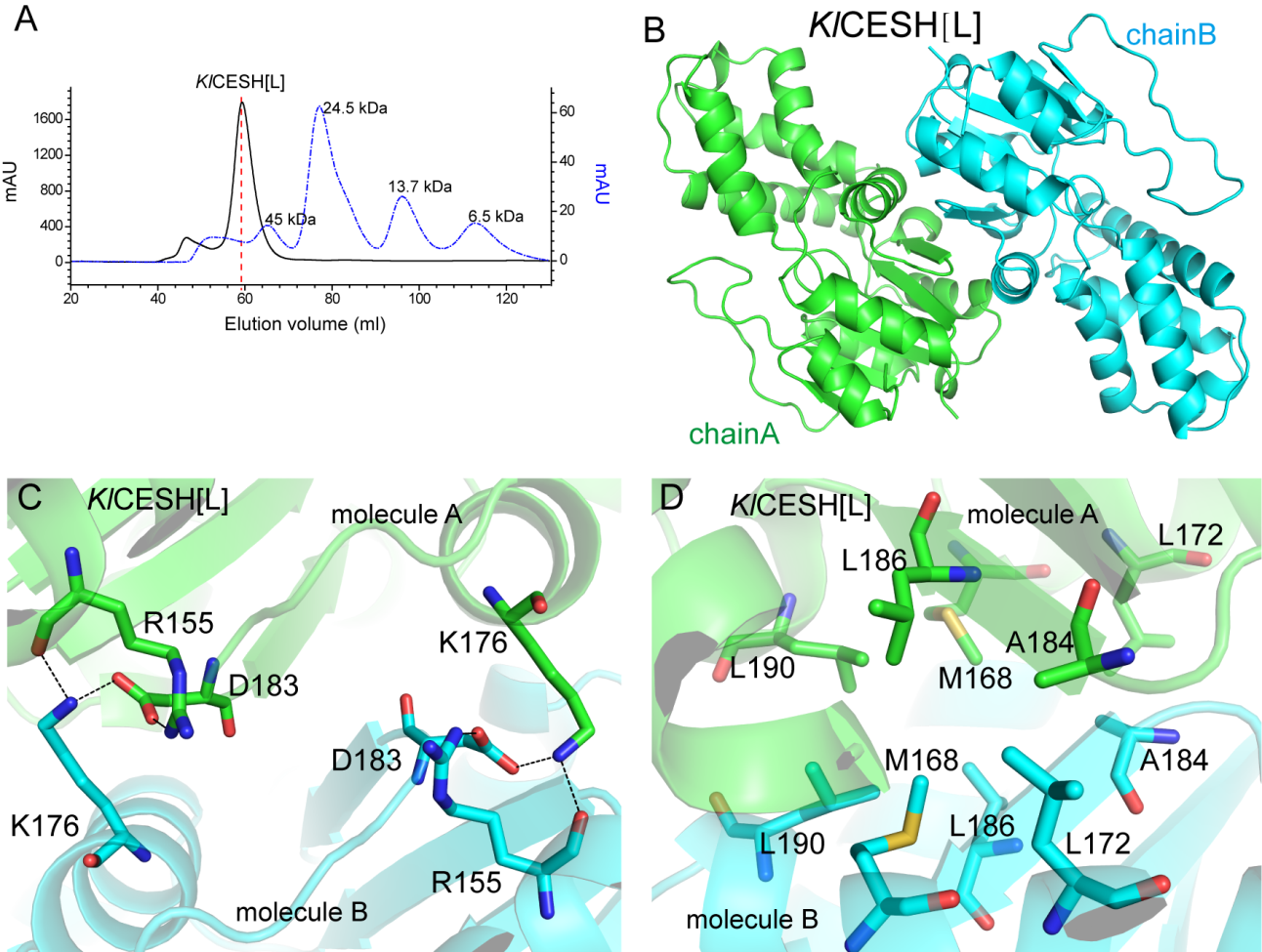


**Figure S2** Analysis of the dimerization interface of the *Kl*CESH[L] homodimer. A, gel filtration analysis of *Kl*CESH[L]. The theoretical molecular weight of recombinant *Kl*CESH[L] is 27.3 kDa, while its calculated apparent molecular weight is approximately 52 kDa. The solid curve is the result of the gel filtration of *Kl*CESH[L] and the dashed curve is the result of the gel filtration of a molecular standard mixture containing ovalbumin (45.0 kDa), chymotrypsin (24.5 kDa), ribonuclease A (13.7 kDa), and aprotinin (6.5 kDa). B, crystal asymmetric unit consisting of two molecules of *Kl*CESH[L] in green and cyan, respectively. C, analysis of key residues involved in hydrophilic interactions in *Kl*CESH[L] dimerization interface. D, key residues involved in hydrophobic interactions in *Kl*CESH[L] dimerization interface.


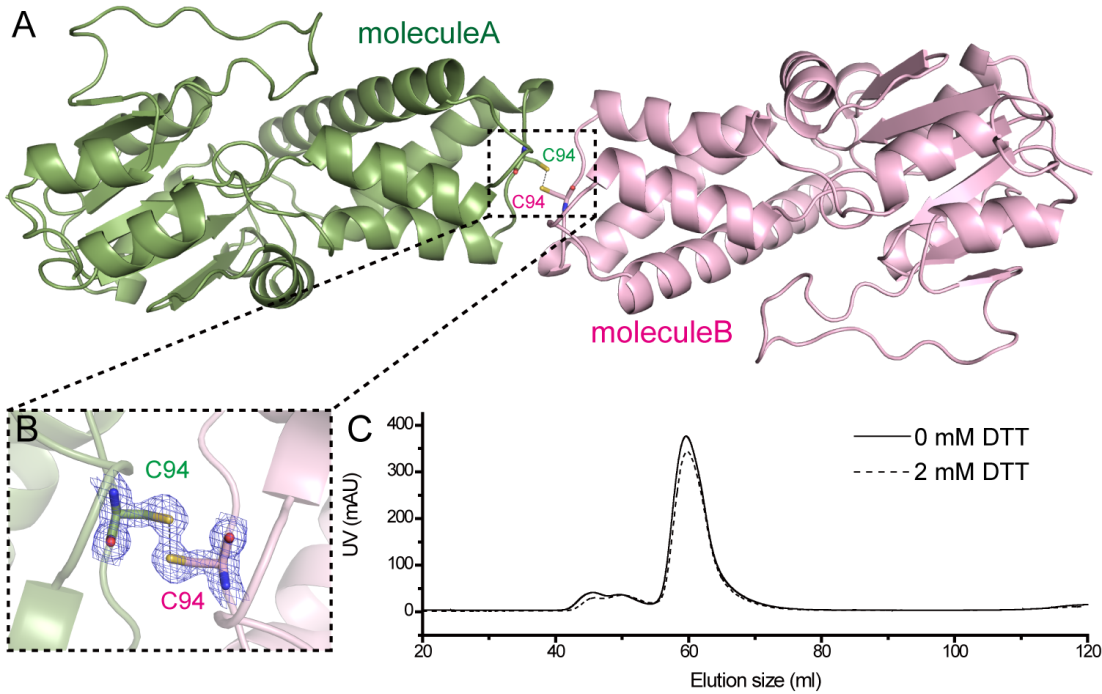


**Figure S3.** The intermolecular disulfate bond formed in the crystal packing of *Rh*CESH[L]. A, the C94 from two neighbor molecules forms a disulfide bond. B, the 2*mF*_o_-*DF*_c_ densities for C94 are shown as blue mesh at 1.0-σ level. C, gel filtration analysis of *Rh*CESH[L] with (dashed curve) and without (solid curve) 2 mM DTT in the buffers.


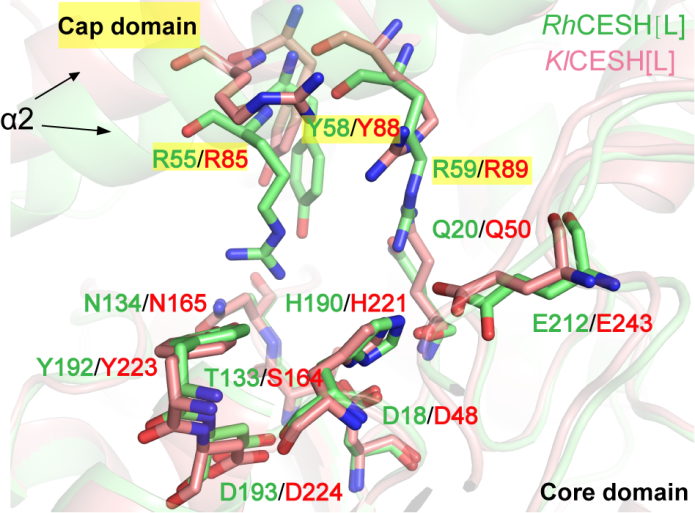


**Figure S4.** Superimposition of residues at the active site of *Rh*CESH[L] and *Kl*CESH[L]. Residues in *Rh*CESH[L] are shown in green and *Kl*CESH[L] are shown in red, respectively, and residues from the cap domain are labeled in yellow shade.


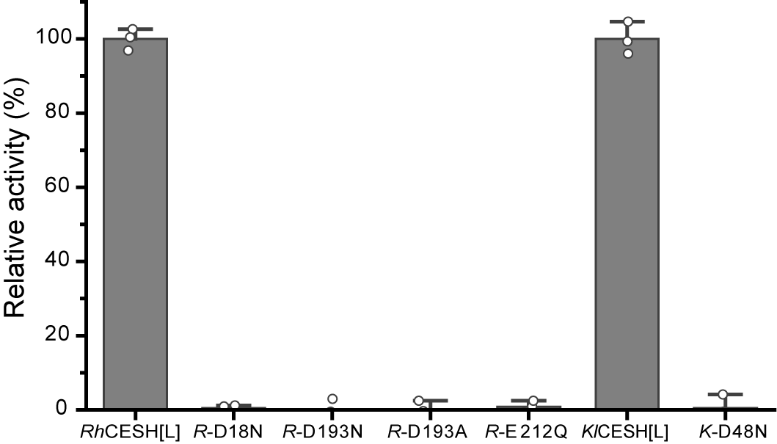


**Figure S5** Relative activities of wild-type CESH[L] and their mutants. The open circles indicate the individual data points of triplicate experiments.


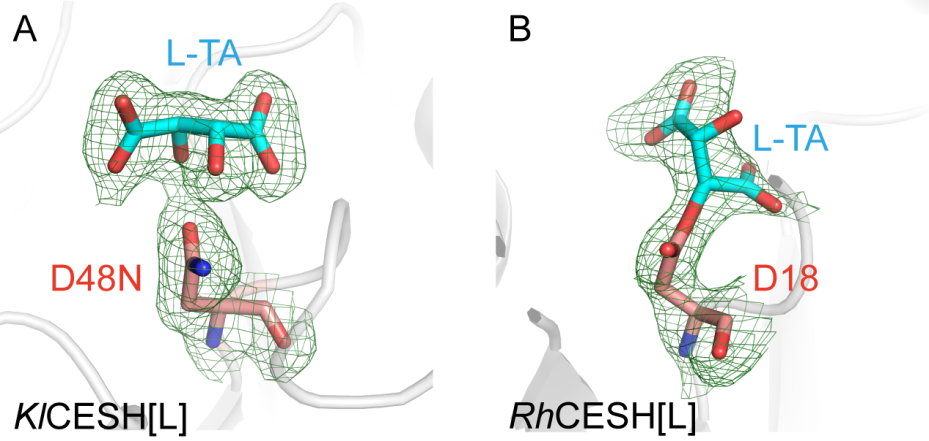


**Figure S6** Composite omit maps of the active site of *Kl*CESH[L]-D48N (A) and *Rh*CESH[L]-E212Q (B). The composite omit maps contoured at 1.0-*σ.*


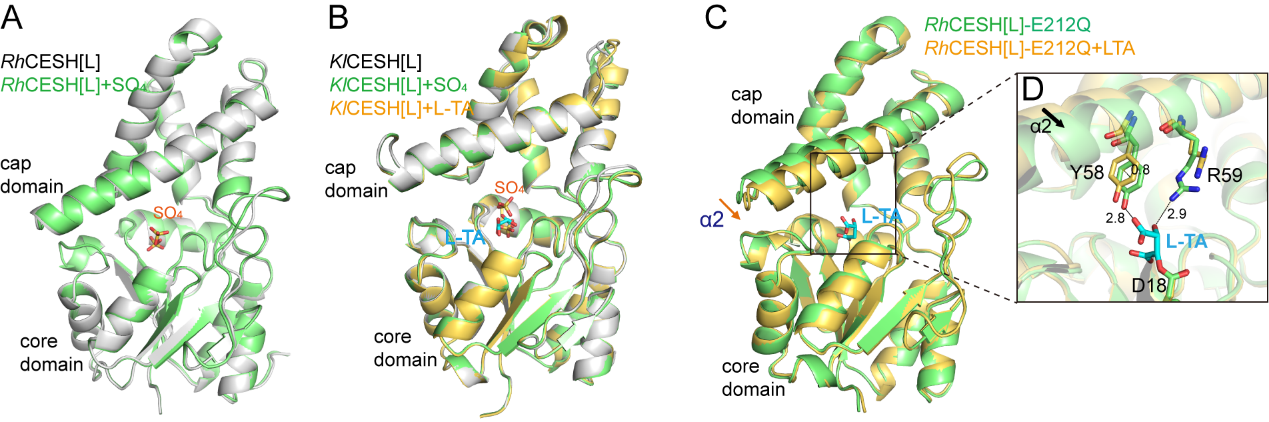


**Figure S7.** Comparative analysis of the impact of ligand binding on the overall structure of CESH[L]. A, structural comparison of *Rh*CESH[L] and *Rh*CESH[L] complexed with sulfate ions. B, Superimposed *Kl*CESH[L], *Kl*CESH[L]/sulfate ions, and *Kl*CESH[L]/L-TA. C, Structural comparison of *Rh*CESH[L]-E212Q and *Rh*CESH[L]-E212Q/L-TA. D, Detailed view of the structural changes caused by binding of substrate. The structures of CESH[L] are coloured to match their respective labels.
